# An integration framework for linking avifauna niche and forest landscape models

**DOI:** 10.1371/journal.pone.0217299

**Published:** 2019-06-07

**Authors:** Eric S. Walsh, Tara Hudiburg

**Affiliations:** Department of Forest, Rangeland, and Fire Sciences University of Idaho, Moscow, Idaho, United States of America; University of British Columbia, CANADA

## Abstract

Avian cavity nesters (ACN) are viable indicators of forest structure, composition, and diversity. Utilizing these species responses in multi-disciplinary climate-avian-forest modeling can improve climate adaptive management. We propose a framework for integrating and evaluating climate-avian-forest models by linking two ACN niche models with a forest landscape model (FLM), LANDIS-II. The framework facilitates the selection of available ACN models for integration, evaluation of model transferability, and evaluation of successful integration of ACN models with a FLM. We found selecting a model for integration depended on its transferability to the study area (Northern Rockies Ecoregion of Idaho in the United States), which limited the species and model types available for transfer. However, transfer evaluation of the tested ACN models indicated a good fit for the study area. Several niche model variables (canopy cover, snag density, and forest cover type) were not directly informed by the LANDIS-II model, which required secondary modeling (Random Forest) to derive values from the FLM outputs. In instances where the Random Forest models performed with a moderate classification accuracy, the overall effect on niche predictions was negligible. Predictions based on LANDIS-II simulations performed similarly to predictions based on the niche model’s original training input types. This supported the conclusion that the proposed framework is viable for informing avian niche models with FLM simulations. Even models that poorly approximate habitat suitability, due to the inherent constraints of predicting spatial niche use of irruptive species produced informative results by identifying areas of management focus. This is primarily because LANDIS-II estimates spatially explicit variables that were unavailable over large spatial extents from alternative datasets. Thus, without integration, one of the ACN niche models was not applicable to the study area. The framework will be useful for integrating avifauna niche and forest ecosystem models, which can inform management of contemporary and future landscapes under differing management and climate scenarios.

## Introduction

The structure and composition of forest ecosystems are expected to shift with climate-induced changes in precipitation, temperature [1], fire [2], carbon mitigation strategies [3,4], and biological disturbances [5]. Specifically, climate change induced declines in tree species occurrence [6], shifts in forest carbon stocks [1], increases in forest mortality events [7], and increases in forest burn area [8] have been predicted. Forest composition and structure are integral to biodiversity [9], and the climate induced changes are likely to have wildlife biodiversity implications [10] especially for avifauna [11]. For example, moderate to high severity fires can create open forest habitat, adequate snag density, and minimal mid-story vegetation for primary avian cavity nesters such as woodpeckers [12]. Though climate models predict increases in area burned or fire intensity, which may increase habitat suitability for woodpeckers [13], tree species composition shifts via climate change may pose adaptation constraints on them [14]. Integrating the feedbacks between wildlife, habitat, and forest processes in a modeling framework could improve our understanding of climate induced wildlife habitat changes and subsequent climate-forest adaptive management.

**Fig 1:**
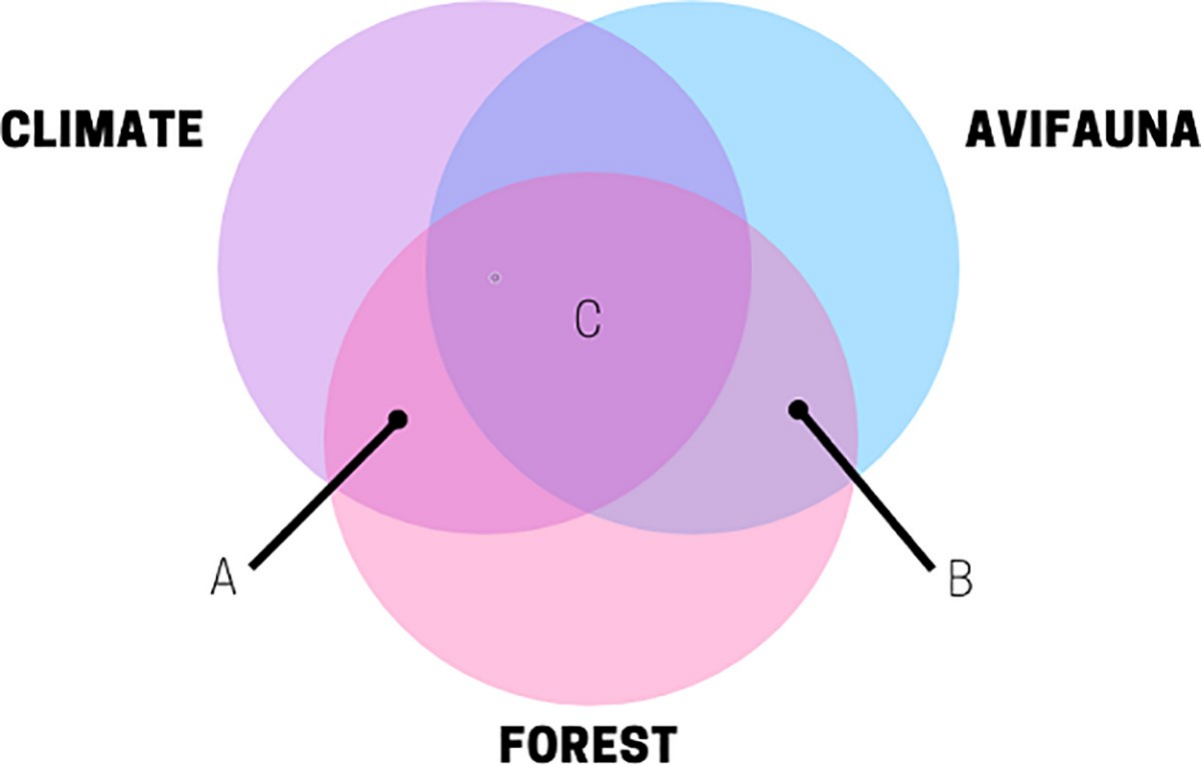
The conceptual diagram of climate-avifauna-forest model integration. A) Spatially explicit forest landscape models with dynamic ecosystem processes that modulate processes via dynamic climate integration like LANDIS-II; B) Avifauna-Forest models that integrate with Climate-Forest models and are not constrained by transferability to novel regions; C) the integration of two different model types to produce emergent results that accounts for climate, vegetation, and avifauna responses. Figure adapted from [15].

A model integration approach that links future forest structure and composition (from mechanistic based forest ecosystem models) with ecological niche models would account for the intrinsic feedbacks between climate, disturbance, and vegetation, and subsequent effects on wildlife (Fig 1). Avian cavity nester (ACN) ecological niche models, both primary excavators and secondary cavity users, are suited for such model integration [15]. ACNs are an ensemble of wildlife species that can function as indicators of forest wildlife biodiversity and ecosystem function. They are suitable indicator species of forest ecosystem dynamics [16–19], because they are ecologically constrained by landscape scale forest components such as composition, structure, disturbance regimes, and management activities. Primary cavity nesters are also correlated with forest avifauna community diversity [16] and cavity nesting webs [20,21]. Some woodpeckers and owls are associated with the characteristics of mature and structurally complex forests [16,22,23], which sustain greater biodiversity [24] and modulate their population responses. These characteristic include snag density [25,26], tree density and diameter [27], burn severity [12,28], and beetle outbreaks in the western North America [29]. These forest ecosystem components will be impacted by climate change [5,30,31] having cascading effects on ACNs responses rendering them viable indicators in modeling future changes to forest ecosystems under a range of climate and management scenarios.

The integrated approach would have two primary outcomes. First, the inclusion of vegetation and other ecological constraints can further improve climate change based avian distribution models [32,33]. The integration of vegetation responses (i.e., habitat component) into an avifauna distribution model framework via dynamic global vegetation models (DGVM: models that project vegetation type shifts) has been shown to be effective at modeling avifauna responses to climate change [34]. Moreover, fine-scale vegetation modeling of specific environments (e.g., montane and boreal environments) [35,36] or for species with narrow habitat breadth [37] may be necessary to feasibly model avifauna distributional changes under a changing climate. Integrating process-based forest landscape models such as the LANDIS models (LANDIS-II and LANDIS PRO) that incorporate finer scale climate-vegetation-disturbance interactions is promising [38–41]. Many of the key habitat characteristics and processes (e.g., forest composition and structure; disturbance type, intensity, and temporal trends) that modulate ACN habitat use responses are output variables of forest landscape models, allowing for points of integration between the two modeling disciplines.

Second, the projected avifauna responses after integration provide an additional metric beyond biogeochemical to assess differing future scenario effects (e.g., climate, management, and natural disturbance) on forest ecosystems. Some studies that have used this integration approach with ACNs (Fig 1) found that managing for forest carbon storage decreased suitable habitat [41] and avifauna populations would decline under climate change or business as usual timber harvest practices [40]. Despite these previous efforts, model integration has been limited to a few studies [38] and is hampered by lack of a framework for transferring ecological niche models, especially realized niche models.

We present a framework to integrate ACN ecological niche models with a forest landscape model (LANDIS-II) and formalize the process. First, our focus is on using existing models and readily available data to achieve model integration. Since this results in using models that are not trained in situ, our first objective is to address transferability. Second, because forest landscape model outputs do not always function as direct inputs into ACN ecological niche model (e.g. percent forest cover versus age or leaf area index), our second objective is to explore the methods necessary to translate forest landscape model outputs into the inputs required. Our final objective is to address the process of verifying that forest landscape model outputs will adequately inform an ecological niche model when compared to the niche model’s original inputs (i.e., the input types used to originally train the niche model). We use the standardized terminology of ecological niche model, realized niche model, potential niche model, and habitat suitability map proposed by [42].

## Materials and methods

### Study area

The study area used to test the framework was the Environmental Protection Agency Level III Northern Rockies Ecoregion of northern Idaho (47.863437, -116.343855) [43]. This area covers 3.1 million hectares and is 88% forested. The region is 61% publicly held with 76% of the public land managed by the U.S. Forest Service. It is comprised of ponderosa pine (*Pinus ponderosa*), lodgepole pine (*Pinus contorta*), Douglas-fir (*Pseudotsuga menziesii*), western larch (*Larix occidentalis*), western white pine (*Pinus monticola*), western red cedar (*Thuja plicata*), grand fir (*Abies grandis*), and western hemlock (*Tsuga heterophylla*). The climate varies across topographic gradients from cooler-wet to warmer-dry affecting forest productivity and fuel loads. Historically, the region had a mixed-severity fire regime with low/moderate severity fire rotations of < 20 years in the low to mid-elevation forests [44] to high severity fires occurring every 150–500 years across elevation gradients [45,46]. The similarity of the study area to other Intermountain West forest ecosystems because of a mosaic of forest cover types due to topographic, climatic, and disturbance heterogeneity was advantageous for a study dependent on the transfer of western avian cavity nesting species ecological niche models.

### Model integration framework

#### Models integrated

**Fig 2:**
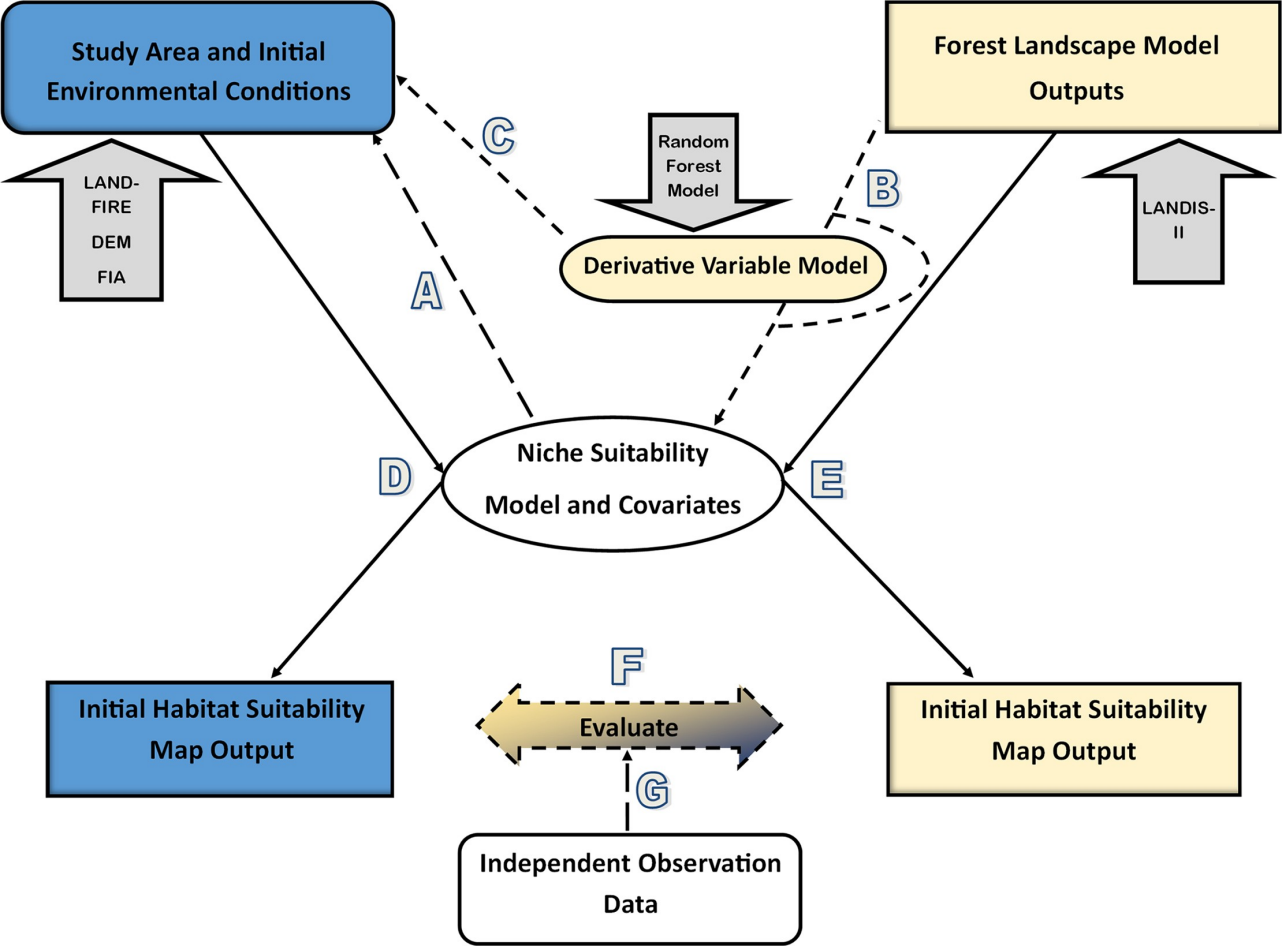
The framework for linking existing niche suitability models with forest landscape models to achieve integration. The left side represents the pathway (D) of estimating habitat suitability using existing ecological niche models and enviornmental conditions of the study area independent of a forest landscape model. The right side represents the pathway (E) of estimating habitat suitability using a forest landscape model (here: LANDIS-II) with integration points. The pathways and integration points are described in the Model Integration Framework section. Shape key: ovals are models; right-angle rectangles are outputs of models; rounded edge rectangles are environmental conditions in the study area; dashed lines represent steps that involve evaluation processes; solid lines represent implementation; block arrows represent data/models used in this study.

Our first objective of model transferability and second objective of linking the niche model outputs with LANDIS-II imposed constraints on the overall pool of candidate models. Taxonomically, bird species distribution models transfer better to other regions compared to invertebrate and plant models [47]. However, the transferability of a model both spatially and temporally requires an evaluation of environmental equilibrium of the species, i.e., the species occurs in all climatically suitable areas and is absent from those that are unsuitable [48], environmental similarity between model training and projection regions, and maintenance of the correlation matrix among predictors between regions [49]. These constraints and others related to model development methods [50] limit the number of models available for integration, because models that pertain to a specific focal species, region, temporal period, transferability potential, and accommodate the level of inference desired are not readily available. Thus, the availability of models for this study was predominantly limited because of: 1) transferability issues due to the inability to apply a model to a novel landscape (e.g., machine learning models (e.g., MAXENT) do not produce parametric equations for publication easily, hampering model application beyond the training region) (Fig 2A) or; 2) the lack of parameter concordance between the LANDIS-II outputs and avian model inputs or its derivatives (Fig 2B). It is unfeasible to report on each model rejected for integration because of the failure of transferability or linking, therefore we present the models that fit the selection criteria and then discuss the general reasons for model rejections.

We present two avian niche models to demonstrate the process of model integration with LANDIS-II. The first was a Flammulated Owl (*Psiloscops flammeolus*) realized niche model that predicts potential distribution and was originally informed by presence-absence data while accounting for imperfect detection [51]. The Flammulated Owl model was trained in an Intermountain West ecological region similar to our study area, which allowed for transferability. The second was an American Three-toed Woodpecker (*Picoides dorsalis*) potential niche model that predicts the extent of suitable habitat without considering potential distributions [52]. The American Three-toed Woodpecker model is considered broadly applicable across the woodpecker’s distribution [52], thus rendering it readily available and transferable. Both avifauna niche models resulted in habitat suitability maps of the study region. We selected these models for pragmatic reasons, because they met the transferability criteria and integrated with LANDIS-II. Further, they demonstrate the application of realized and potential niche models within the framework.

The forest landscape model implemented was LANDIS-II (v.6.2.1), which has a library of extensions that facilitate the simulation of multiscale ecosystem processes with spatial interactions and dynamic communities at scales of 10^4^−10^7^ ha [53]. These multiscale processes and dynamics are simulated at variable timesteps within an interacting gridded landscape with each cell representing aggregates of species-age cohorts and respective biomass. Twelve tree species and ecosystem processes were simulated at a grain resolution of 200 m (4 ha). Several methods have been used to initialize landscape conditions [54,55], the species-age cohorts across the simulated study area were imputed using Forest Inventory and Analysis (FIA) data and Landscape Builder [56], which is a program that develops a spatially representative landscape from FIA data. Biomass totals are then assigned to each grid-cell through a spin-up process in LANDIS-II, which results in landscape initial conditions.

Successional processes were modulated using the Net Ecosystem Carbon Nitrogen extension (NECN) (v.4.2.4) (formally the Century Succession extension) [57] to model biogeochemical responses and ecosystem fluxes. NECN is based in part on the globally utilized CENTURY soil model [58] and simulates the establishment, growth, and mortality of species, accumulation and decomposition of wood and litter, soil carbon pools, and water availability [57,59]. Disturbance processes (fire and timber harvest) were simulated using the Dynamic Fire and Fuels System (DFFS) and Biomass Harvest. The DFFS extension (v.2.0.3) is based on the Canadian Wildland Fire Information System and modulates fire events based on fuel a, fire-weather, ignition probability, and topography [60]. Biomass Harvest (v.3.2) simulates multiple harvest prescriptions across differing scales [61]. All extensions (succession and disturbance) were simulated at five-year time intervals, such that timestep-0 refers to spin-up initial conditions and timestep-1 refers to the first simulation step, i.e., five years of growth and disturbance interactions. The outputs of spin-up and timestep-1 of the LANDIS-II model were used as direct or indirect inputs into the avifauna niche models (Tables 1 and 2).

**Table 1:** The variables used to inform the Flammulated Owl realized niche model reported by [51].

Parameter	Original (*base model)* Input Source	LANDIS-II Model Source	Original Model Coefficient (logistic)
Aspect (cosine)	Digital elevation model derivative	Digital elevation model derivative	-2.544
Canopy	LANDFIRE Forest Canopy Cover [65]	A Random Forest model of canopy cover based on biomass estimates.	0.064
Diversity	Shannon Diversity Index of the LANDFIRE Existing Vegetation Type classes of the study area	Shannon Diversity Index of the Random Forest modeled cover types.	-1.209
Douglas-fir	LANDFIRE Existing Vegetation Type [65]	Proportion of Douglas-fir from the Random Forest modeled cover types.	0.994
Non-forest	LANDFIRE Existing Vegetation Type [65]	Proportion of non-forest from the Random Forest modeled cover types.	-0.021
Ponderosa pine	LANDFIRE Existing Vegetation Type [65]	Proportion of ponderosa pine from the Random Forest modeled cover types.	0.013

**Table 2:** The variables used to inform the American Three-toed Woodpecker potential niche model reported by [52].

Parameter (Indicator)	Value	LANDIS-II Model Source
Tracts of old-growth forest	Continuous tracts of land with a cumulative area > 1,000 km^2^ (*very good)*; area 600–1000 km^2^ (*good*)	First simulated timestep: all continuous grid cells with an average forest age > = 125
Plant associations*	Spruce (*Picea* spp.) forest (*very good*); Spruce and Mixed-conifer/Douglas-fir (*good*)	The area of forest cover resulting from the first simulated timestep. The forest type dataset was the same as the Flammulated Owl model.
Stand age*^+^	> = 125 yrs (*very good*) > = 90 yrs (*good*)	First simulated timestep: all grid cells with an average forest age meeting the criteria
Snags and decadent trees, especially those with heart rot*^++^	>1.2 snags/ha (this fulfills *very good* and *good* levels)	First simulated timestep: a binary variable predicted using a Random Forest model with live above ground biomass, average forest age, elevation, slope, and aspect as predictor variables.
Natural forest disturbance*	Disturbed < = 5 years (this fulfills *very good* and *good* levels)	First simulated timestep: burned grid cells
Elevation*^+++^	4300–9000 ft	DEM
Timber Harvest*	Rotations > 100 yrs. (this fulfills *very good* and *good* levels)	First simulated timestep: all grid cells with an average forest age meeting the criterion

* Ecological indicators that are considered important indicators

+ The stand age was parameterized as a categorical value (old-growth and mature) in [52]. We associated a value of 125 years from a reference in [40].

++ The snag density was parameterized as a categorical value (abundant) in [52]. We associated a value of 1.2 snags/ha based on American Three-toed Woodpecker habitat suitability model [70].

+++ The elevation proposed by [52] was specific to Utah. The best information on elevation gradients in Idaho indicate mid-elevation habitat use and across the American Three-toed Woodpecker western distribution an elevation range of 4300’– 9000’ [71].

#### Transferability assessment

The transferability assessment of the avifauna niche models (Fig 2A) was implemented following the suggestions of [49]. The first assumption of transferability is that the species is in equilibrium with the environment or current climate suggesting that the species occupies all climatically suitable habitat. This can be evaluated by comparing the observed distributions to modeled distributions based on climate envelope modeling or through covariation analysis between species assemblages and climate [62]. The latter analysis indicates birds have a high covariation with climate and can be assumed to be at equilibrium, which is likely because of dispersal ability [62]. Thus, we accepted the assumption of equilibrium for the Flammulated Owl and American Three-toed Woodpecker without additional analyses.

The second assumption of transferability is the study area and training region have similar environmental characteristics. We tested for similarity between the study area and training regions by comparing the distribution of each model’s predictors using the multivariate environmental similarity surface (MESS) methods outlined in [63] and [64]. In our study, the MESS calculates how similar a grid cell in the study area is to the set of grid cells in the training region based on the set of predictors of the respective model being evaluated. As a grid cell approaches 100, the location is less novel because the study area predictor values are approaching the median value in the training area. A negative cell value indicates a predictor in the study area that is outside the range of the training area, i.e., the cell represents a novel environment (see MAXENT Novel tutorial at http://biodiversityinformatics.amnh.org/open_source/maxent). The third assumption of transferability is the covariation structure of the predictor variables remains spatially and temporally constant between the study area and training regions. We tested for changes in the correlation matrix using a Pearson correlation coefficient. We report on the evaluation of the latter two assumptions, environmental similarity and predictor variable covariation structure.

### Integration process

#### Flammulated owl model

The Flammulated Owl model was originally parametrized (hereafter referred to as the *base model*) and trained using data from the Boise National Forest in southern Idaho. The input variables (Table 1) were processed for our study area at the appropriate spatial scales reported in the original study. An *initial* habitat suitability map (initial probability of occupancy) was calculated for our study area using the same methods, data, and state variable types described in [51], i.e., *base model* (Fig 2D). This initial habitat suitability map was used to evaluate the efficacy of using LANDIS-II to inform the Flammulated Owl model (Fig 2F), by comparing this *base model* map to the habitat suitability maps generated using LANDIS-II outputs and modeled canopy cover, modeled land cover types, and both.

The parameters of the Flammulated Owl model were not all directly informed by the LANDIS-II outputs; canopy cover and land cover required secondary modeling (derivative variable model) (Table 1) (Fig 2B). The *base model* uses the LANDFIRE Forest Canopy Cover dataset [65] aggregated into four canopy cover classes (1 = 0–10%, 2 = 11–40%, 3 = 41–70%, and 4 = 71–100%) with the category’s midpoint value assigned to a grid cell. To calculate canopy cover from LANDIS-II outputs, we used Random Forest (RF) [66] to predict the canopy cover classes of the study area from the LANDIS-II biomass estimates (S1 File). To evaluate the secondary modeling process, we first used the RF classification prediction error (Out-of-Bag (OOB)) to approximate the model’s internal performance at predicting the observed canopy cover (Fig 2C). OOB is the prediction error of a RF model derived internally through a bootstrapping process of sub-sampled data. Second, we evaluated the sufficiency of the RF canopy cover predictions from LANDIS-II simulated state variables at timestep-0 to adequately model an initial habitat suitability map by comparing it to the suitability map informed by LANDFIRE canopy cover estimates (Fig 2F). We compared the two habitat suitability maps using an ArcGIS 10.5 band collection statistic correlation matrix. The band collection statistic is a multivariate analysis of raster bands, where in this case the correlation between two raster cells was calculated based on the covariance between the two datasets divided by the product of the standard deviations.

Land cover types and diversity (Shannon Diversity Index) metrics of the Flammulated Owl *base model* are based on the land cover classes of the LANDFIRE Existing Vegetation Type (EVT) within differing buffers around each grid cell. The *base model* is parameterized using 11 EVT classes [51]. The LANDIS-II internally classified forest types (based on Biomass Reclass Output v.2.0) of initial spin-up conditions based on the Landscape Builder extrapolation of FIA data resulted in much more spatial heterogeneity of forest cover types than the LANDFIRE classifications for the study area. This is likely due to the homogeneous nature of LANDFIRE EVT classifications. This required a RF model to spatially predict the LANDFIRE cover types of the initial landscape from the LANDIS-II species composition-biomass spin-up values (S1 File). Like the canopy cover model, the OOB (Fig 2C) and comparison between the two initial habitat suitability maps (Fig 2F) (one informed with the LANDFIRE EVT cover types and one with the LANDIS-II RF cover types) were used to evaluate the secondary modeling process.

Finally, we generated habitat suitability maps based on the probability of occupancy without aggregating into discrete suitability levels, as this tends to diminish the available information [67]. We compared (Fig 2F) the habitat suitability map informed by the *base model* datasets (Fig 2D) to the LANDIS-II and derivative variable models informed habitat suitability map (Fig 2E) using the ArcGIS 10.5 Band Collection Statistic correlation matrix. We assumed in all instances that the habitat suitability map generated from the *base model* (Fig 2D) represented the study area, and the LANDIS-II informed habitat suitability map with secondary modeling was being evaluated (Fig 2E). Observed Flammulated Owl location data from the Idaho Department of Fish and Game (IDFG) [68] and an associated 400 m buffer representing a home-range [51] were also compared to the occupancy predictions (Fig 2G).

#### American three-toed woodpecker model

The American Three-toed Woodpecker potential niche model applied here was developed by the Utah Division of Wildlife Resources and The Nature Conservancy using an Ecological Integrity Table (EIT) format [52]. EITs identify the key ecological attributes or conceptual factors (e.g., environmental regimes and constraints) that sustain a target’s (here: a species) composition, natural dynamics, and long-term persistence [52,69]. Associated with the conceptual factors are real indicators that can be quantified or qualified to assess ecological integrity. The transferability assessment was not relevant to this model (Fig 2A), because it is a trait based potential niche model based on threshold assessments of environmental parameters to produce ordinal levels of suitability. The model is explicitly intended to be broadly applicable across the woodpecker’s range (i.e., transferable); the exception being the elevation indicator which is applicable specifically to Utah [52]. Unlike the Flammulated Owl model, which could be informed by the original model development datasets and LANDIS-II outputs, these model characteristics precluded the development of a comparative habitat suitability map for model verification. We therefore assumed the model informed from LANDIS-II outputs was an accurate representation of suitable habitat.

The American Three-toed Woodpecker model has 12 indictors of which eight are considered most important [52]. We included six important indicators and one of the alternative indicators (Table 2) omitting the important indicators *larvae of bark beetles* and *forest management*. This was necessary, because we did not model biological disturbances in the LANDIS-II modeling, and there was no process to identify areas of *none* management activity associated with the EIT’s *forest management*: *very good* suitability level. However, the *forest management* indicator is indirectly included among the other indicators such as *stand age* [52]. Further, it was not possible to explicitly identify non-harvested areas of the previous 100 years to properly inform the *timber harvest* indicator. We used *stand age* as a proxy to identify areas with rotation ages >100 years to meet the criteria of the *timber harvest* indicator (Table 2).

This ecological niche model required simulated state variables from the timestep-1 of the LANDIS-II model (Table 2). The *plant associations’* spruce-fir cover extent was derived using the same land cover RF model as the Flammulated Owl process. We parametrized the *natural forest disturbance* indicator with a binary variable identifying the simulated burn areas in timestep-1. The *elevation* parameterizations were not applicable outside of Utah and were assigned new threshold values based on the study area [71] (Table 2). The *stand age and snag and decadent trees* indicators are qualitative ordinal variables in the EIT. To improve spatial modeling of these variables in a GIS, we assigned quantitative thresholds from other published sources [70,71] (Table 2). *Stand age* was informed from the LANDIS-II timestep-1 landscape maps. S*nag and decadent trees* indicator could not be directly informed by LANDIS-II outputs (Fig 2B). We implemented a RF classification model using FIA data [72] (S1 File) to predict a binary (present/absent) response for the appropriate snag density of each grid cell of the study area, because predicting a quantitative snag density response would be uninformative due to FIA methodology. The FIA methodology for plot level estimates result in a minimum scaled snag density of ~14 snags/ha for each recorded dead tree on a plot, which is significantly above the optimal 1.2 snags/ha associated with the American Three-Toed Woodpecker. For RF model training, we filtered the response variable to only include dead standing trees meeting specific criteria (dbh > 26 cm, height > 21 m, and decay code > 2). By accounting for only snags meeting these criteria, we indirectly accounted for the EIT indicators *dbh of snags* and *height of snags* in our modeling.

The habitat suitability maps were produced by assigning each indicator layer grid cell with the respective condition present a one through seven-digit value with each initial digit being unique to the layer (e.g., layer 1 = 1, layer 2 = 20, layer 3 = 300, etc.). These layers were added together in a GIS to create a final gridded surface representing the potential niche (i.e., at each grid cell from zero to seven layers intersected, the specific combination of intersecting layers was captured at each grid cell) with cells that lacked the inclusion of one or more indicators being explicitly identified. A *suitability index value* representing the number of intersecting indicator layers present at a grid cell was then assigned to a new gridded surface. For example, if grid cell *x* was coded as 7604321, one gridded surface accounted for the layer 5 conditions being absent from cell *x*, and a second gridded surface coded cell *x* with a *suitability index value* of *six*.

Like the Flammulated Owl assessment, observed locations of the American Three-toed Woodpecker [68] were compared to the potential habitat suitability maps by assigning the *suitability index value* to each observation point and summarizing (Fig 2G). To account for the potential territorial habitat around an observation point, we quantified the suitable habitat using a 147 ha buffer. The buffer was based on the median value of the reported highly variable territory sizes [71]. We summarized the percent area of forest land cover associated with each ecological indicator and *suitability index value* depending on the suitability level, and the number of indicators associated with each buffer (majority and minimum).

## Results

### Flammulated owl model

The Flammulated Owl realized niche model was implemented because the transferability assessment suggested it was an acceptable fit for the Northern Rockies Ecoregion of Idaho. The MESS analysis indicated the study area did not have many novel locations compared to the training region; almost all predictor values within the Northern Rockies Ecoregion are within the range found in the training region (S1 Fig). The covariance structure of predictors was mostly consistent between areas; several variable pairs only differed in degree of correlation (S2 Fig). However, the ponderosa pine density variable differed in direction and degree with respect to non-forest land cover density and canopy cover (S2 Fig).

The RF model that predicted canopy cover classes from the LANDIS-II outputs was adequate (OOB accuracy: 54.9%) (S1 File). The model overestimated the grid cells that were considered medium, underestimating the high and low canopy classes (S1 File). However, the overall performance of the RF did not appear to affect the predicted Flammulated Owl distribution; the two distributions (habitat suitability maps comparisons based on the LANDFIRE and RF canopy cover) were 99.6% correlated. This is plausible since the effect of canopy cover on occupancy is minimal (Table 1). The RF model that predicted land cover classes from the LANDIS-II outputs performed well (OOB accuracy: 90.6%) (S1 File). Land cover classes with sparse representation across the study area were predicted poorly. However, of the three land cover parameters that informed the Flammulated Owl model (Douglas-fir, non-forest, and ponderosa pine), ponderosa pine had the highest prediction error rate (43%) (S1 File) and Douglas-fir had the lowest prediction error rate (3%) (S1 File), which is a strong predictor of Flammulated Owl habitat occupancy (Table 1).

**Fig 3:**
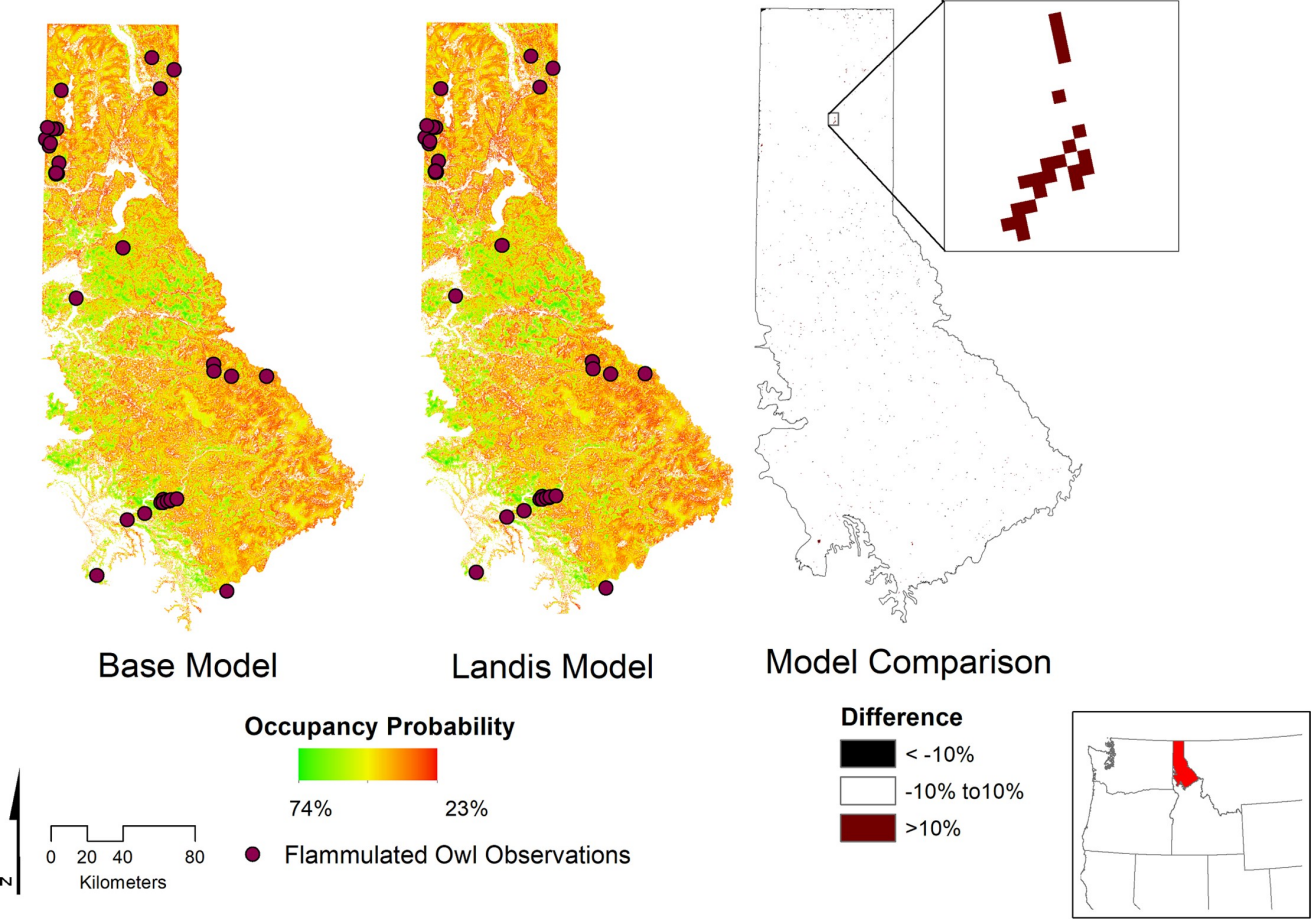
The contemporary habitat suitability map of the flammulated owl across the Northern Rockies Ecoregion of Idaho with observed locations. The habitat suitability maps were based on the occupancy probability using the realized niche suitability model described in [51]. Two data sources were used to inform the niche model: original sources as described by [51] (Base Model) and inputs sourced from the LANDIS-II forest landscape model (Landis Model). Differences greater than +/- 10% between models informed with the differing input sources are too slight to be visible (inset map).

The predicted realized niche habitat suitability map using the LANDIS-II outputs and derivative variable models (RF canopy cover and RF land cover) was 94.8% correlated with the *base model* predictions (Fig 3) (Note: this was the same correlation observed in the RF land cover and LANDFIRE canopy cover modeled habitat suitability map compared to the *base model*, because RF canopy cover estimates had little effect on the final habitat suitability maps when compared to the implementation of LANDFIRE canopy cover). Differences >10% in occupancy probability were negligible, being mostly relegated to the edges of the study area and non-forested areas (Fig 3). The probability of occupancy was similar between the *base model* and LANDIS-II habitat suitability maps, in addition to the observed Flammulated Owl locations among both maps (Table 3) (S3 Fig). Among the observed Owl locations, the probability of occupancy was low (Table 3), however the habitat buffers of the known locations did contain older forest stands (mean = 93 years old).

**Table 3:** The summary statistics of the *base model* and LANDIS-II Flammulated Owl occupancy probability predictions of the study area with the observation point data.

	Habitat Suitability Comparison		Observation Location Comparison		
	Base Model	LANDIS-II	Base Model	LANDIS-II	LANDIS-II Buffers
Mean	43%	43%	47%	46%	44%
Minimum	23%	23%	34%	33%	37%
Maximum	74%	74%	62%	62%	51%
Count	NA	NA	28	28	28

### American three-toed woodpecker model

**Fig 4:**
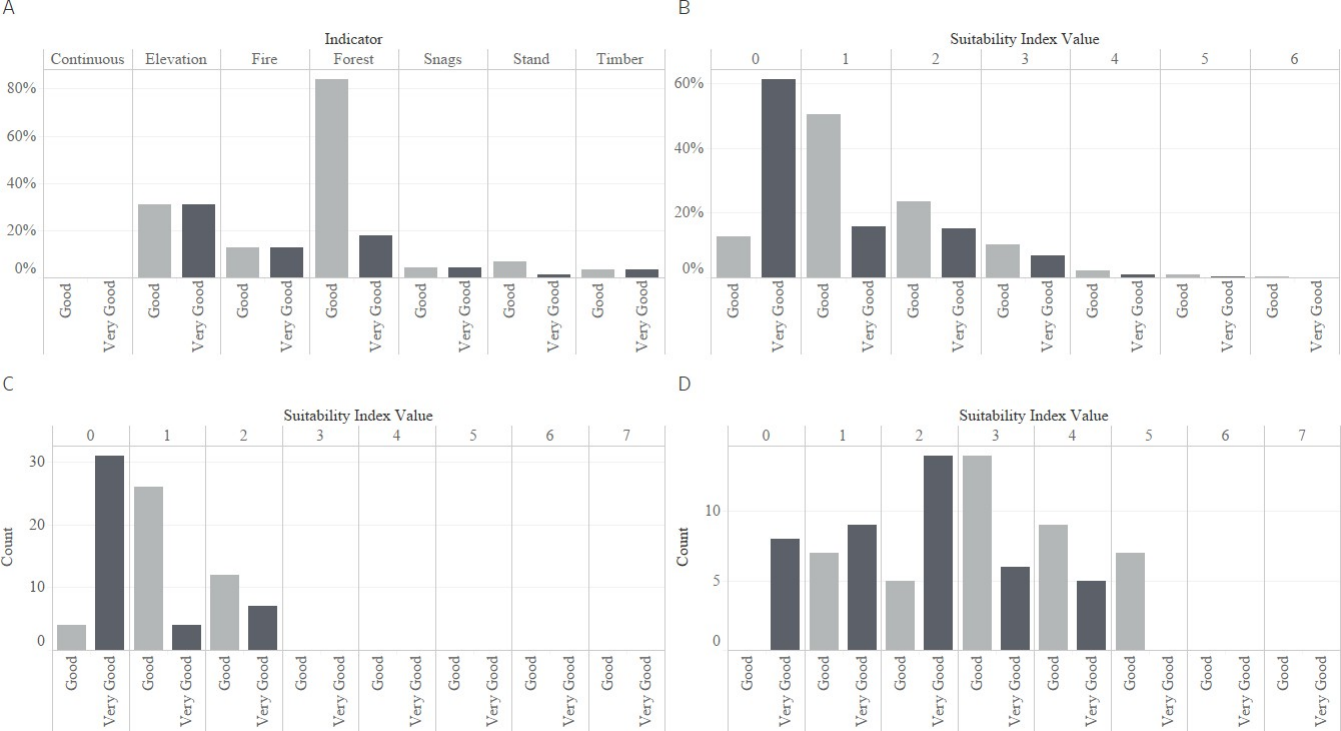
Among the American Three-toed Woodpecker *very good* and *good* habitat suitability models: (A) is the percent of forest land cover associated with each ecological indicator (these can sum to >100 because areas can be associated with more than one indicator); (B) is distribution of forest land cover associated with each *suitability index value*; (C) is the count of observation point buffers that have a majority of grid cells coded with the specific *suitability index value*; (D) is the count of observation point buffers that contain a minimum of one grid cell with the respective *suitability index value*.

The EIT was not fully informed because the study area lacked contiguous blocks of mature or old-growth forest that met the *tracts of old-growth forest* indicator threshold (Fig 4A). Most of the potential niche was associated with public lands, specifically the U.S. Forest Service. Both levels of suitability were limited by the area of appropriate stand age, timber rotation, and snag presence (Fig 4A). The snag RF model (S1 File) was moderately sufficient (OOB accuracy: 74.3%) to predict presence across the landscape. Less than 2% (*very good*) and 4% (*good*) of the region was associated with four or more of the EIT indicators (Fig 4B) with 1476 ha of *very good* and 5544 ha of *good* suitable habitat associated with six indicators.

**Fig 5:**
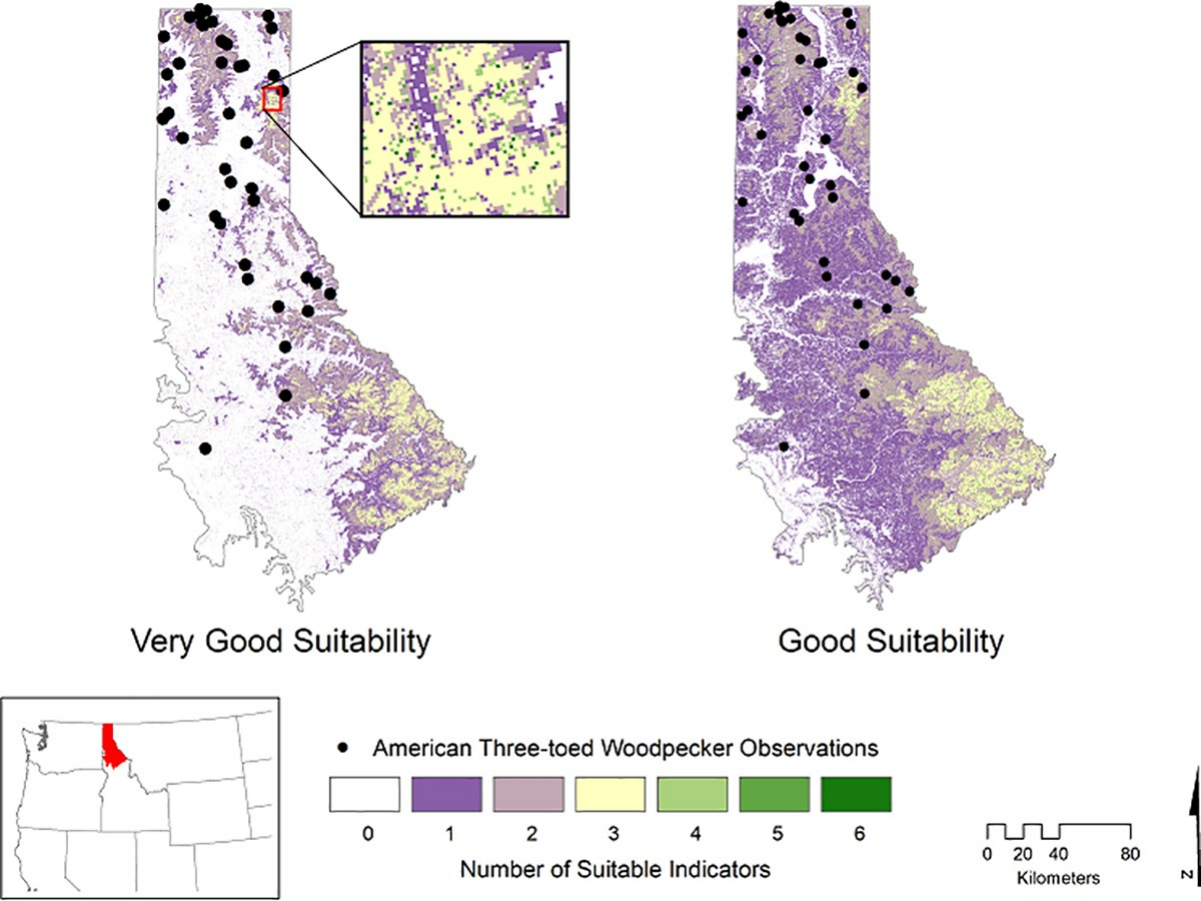
The number of American Three-toed Woodpecker potential niche ecological indicators intersecting across the Northern Rockies Ecoregion for two habitat suitability levels. Expanded inset map depicts the disjunct areas of increased suitability.

Areas with at least three indicators present comprised major contiguous tracts of potential suitability. Areas with more than three indicators present were disjunct and sparsely distributed across the landscape (Fig 5). There was a slight increase in area of suitability level *good* because of the inclusion of mixed-conifer and Douglas-fir cover in the *plant associations* indicator. However, this had minimal impact on increasing the area of suitable habitat based on the total number of indicators present (Fig 4B). At the *very good* and *good* suitability level, the observed American Three-toed Woodpecker point habitat buffers were mostly associated with areas relegated to no indicators and one indicator present, respectively (Fig 4C). Some buffers contained areas with up to five EIT indicators (Fig 4D). The observed Woodpecker locations had a mean elevation of 1060 m, majority mixed-conifer land cover, median stand age of 52, and were subjected to a simulated burn. A comparison of FIA plots meeting the *very good* and *good* suitability level to the predictions indicated spatial agreement (S4 Fig). The FIA plots were not located outside the areas predicted to have one or more EIT indicators.

## Discussion

Ecological modeling focused on the effects of climate change and management scenarios (e.g., fire, carbon mitigation, harvest) on forest resiliency will need to account for the effects of these dynamics on wildlife habitat. Thus, to implement climate adaptive management strategies aimed at increasing or preserving wildlife species, modeling efforts will need to include the coupled response of vegetation *and* wildlife to climate change. We evaluated a framework for integrating ACN ecological niche and forest landscape models to improve ACN climate change niche modeling and provide ecological modelers with a means to account for wildlife measures in biogeochemical forest modeling.

### Transferability assessment

Ecological niche models developed independent of forest landscape models will often require a transfer from the model’s trained to implementation region, increasing the uncertainty of model applicability to a potentially novel region. Generally, models are more readily transferable when they capture the foundations of the ecological niche [73] and use predictors that encompass a wider environmental range in the development region that have direct physiologically or resource meaningful associations [74]. To maximize transferability potential, a candidate set of models should be constructed based on the environmental similarity between the training region and study area, the methods used to evaluate model fit within the training region, the ecological rationale and association between predictor variables and niche response, and the type of model (inference focused or machine learning).

Ideally, mechanistic models, which are more robust at capturing the processes that limit species distributions [75] would be implemented within this framework. It will not always be feasible to implement a process-based niche model, since a significant proportion of ecological niche models are correlative models [76] and are readily available for implementation. Therefore, the primary limiting factor of applying the framework is identifying niche models that are trained in geographic and environmental space similar to the study region. This ensures transferred correlative models are applied to environments with similar covariance structures. Meeting this criterion is important because geographical orientation, anthropogenic land use, and ecological memory affect species distributions and are difficult to standardize across regions [74]. This factor limited the implementation of models in this study (e.g., [77]), because the models were developed in regions too dissimilar to the study area.

Transferability is also limited by the niche model algorithm. A Black-backed Woodpecker (*Picoides arcticus*) niche model developed in the same ecoregion but outside the study area [78] met the geographic constraint criterion. However, the model was developed from a machine learning algorithm (MAXENT), which limits transferability. Models that use predictor variables based on sound ecological relationships with close causal links to response variables increase transferability [79]. Ideally then, statistical models are more readily transferable, because they are focused on inference and ascertaining the causal relationships between predictor and response variables that have biological interpretations within clear conceptual frameworks [80]. Alternatively, machine learning processes are more readily used for prediction through identification of patterns in often complex datasets. Ecological interpretations of such models is challenging, because relationships are not readily related to biological knowledge [80]. This makes transferability difficult because though machine learning algorithms like RF are immune to random noise overfitting [66], they are not immune to overfitting due to heterogeneity of predictor-response relationships [81,82]. This overfitting and failure to make general predictions to novel geographic extents has been observed in machine learning processes applied to avifauna distributions [83]. Without a sound ecological basis for modeled predictor-response variable relationships, regardless of the model’s *in situ* prediction success, it is best to avoid transferring these models to a novel region.

The integration framework is intended to support modeling efforts that focus on climate change scenarios and evaluating the temporal changes in avifauna niches. Ensuring the feasibility of a cross-temporal transfer of the niche model is also important; it is best to select models that have been evaluated for parsimony and not just correlative fit. Parsimonious models, those with moderate complexity as determined using for example Akaike’s Information Criterion, exhibit better cross-temporal transferability than models with higher correlative fit to training conditions as determined using Area Under the Curve [82]. Models like the Black-backed Woodpecker niche model previously mentioned with many predictor variables are also limited in transferability because parsimony limits cross-temporal applications.

The Flammulated Owl model applied within this study meets the criteria described. It was developed within a geographic region similar to our study area (S1 and S2 Figs). The model’s predictor variables are based on *a priori* biological knowledge; they approximate known key habitat-use variables and at scales appropriate to capture dynamics of different ecological processes (e.g., juvenile dispersal, predator interactions, and foraging range) [51]. In addition, the training selection criteria to produce a parsimonious model and prevent over-fitting [51] supports its use in a cross-temporal modeling application, i.e., climate change scenario modeling.

Assessing the transferability of a qualitative potential niche model like the American Three-toed Woodpecker model, which is based on ecological integrity assessments, takes a different approach than a realized niche model (Flammulated Owl). The EITs are the tools of an ecological integrity assessment, which evaluate ecosystems for species composition, diversity, and functional organization comparable to that of similar, undisturbed ecosystems in the region [84]. The individual assessments (i.e., EITs) determine the viability of a species within an ecosystem by evaluating the composition, structure, function, and processes occurring within a natural range of variation important for resiliency and adaptation to most natural and anthropogenic perturbations [69]. They are intended among other things to provide a baseline and trend assessment when applied at broad spatial and temporal scales and are inherently transferable [52]. However, differing ecologies across a species’ range and introduced biases because of model development region (i.e., the American Three-toed Woodpecker model was originally developed in Utah and with a western U.S. focus, making transferability to eastern sections of its range questionable) will influence transferability. Unlike the Flammulated Owl model, assessment of transferability required evaluation of which indicators to include, adjusting thresholds to reflect the study region, and transforming indicators to a quantitative form to improve spatial modeling.

The EIT model meets the criteria for selecting a model for inclusion and transferability assessment. First, though originally applicable across the species’ geographic range, the Woodpecker model was developed for a western U.S. state [52]; focusing the model’s indicators to environmental conditions similar to Idaho. Second, the indicators have a close association with the response variable (potential suitable habitat) and are founded on ecological associations, which are inherent features of an EIT [69]. Even with the inherent applicability, model caveats were addressed to improve transferability.

The EIT is constrained by ecological variation across different geographies, correlated indicators, and reliance on taxonomically similar species for ecological information [52]. We addressed these constraints to improve the model’s application to the study area. Environmental variation within the *plant associations* and *elevation* indicators across geographic regions are noted in the EIT. We used the *plant associations* variations in spruce-fir and lodgepole pine/mixed-conifer forest cover to inform suitability levels of *very good* and *good*; thereby expanding the model’s applicability to the study area. In addition, the *elevation* indicator was parameterized based on literature values as this was explicitly noted as being applicable to Utah. Addressing the variations in these indicators improved model transferability to the study area.

We used the correlation among indicators to minimize variable redundancy and account for variables that could not be directly informed. The *stand age*, *timber harvest*, and important but excluded indicator *forest management* were considered correlated (for this study) based on their descriptions in the EIT. We lacked temporally relevant information for *timber harvest and forest management*, which would have affected the transferability and usefulness of the model if unaccounted. Per the EIT, f*orest management* accounts for alterations to the overall natural fire regime, salvaging logging, and suppression logging. Historically, northern Rockies’ forests were intensely managed resulting in forest structural [85] and fire regime changes [86] throughout. The ubiquitous affect across the landscape and lack of spatially explicit historical management information would have resulted in a relegation of the region to the *poor* indicator level for modeling purposes. This would have rendered the *forest management* indicator uninformative for model inclusion. Confounding this was the lack of spatially explicit harvest data to assess *timber harvest* thresholds. The solution was to use the LANDIS-II stand ages at two threshold levels to inform the *timber harvest* and *stand age* (Table 2). This allowed for identification of older stands indicative of long fire return intervals and lack of harvest. As a result, the areas meeting these thresholds were assumed to be “unmanaged” providing a proxy for the f*orest management* important indicator. Though, we implemented solutions to improve transferability and applicability, model evaluation was still a concern.

### Model comparison

The LANDIS-II informed habitat suitability map of the Flammulated Owl model was evaluated against the same niche model informed with an independent dataset (Fig 2F). However, both habitat suitability maps were not completely verified with independent observation data (Fig 2G), since the available observation data was affected by sampling bias. In contrast to the Flammulated Owl model, there was no procedure to quantitatively evaluate (Fig 2F) the American Three-toed Woodpecker model with an alternatively informed model (Fig 2D). The validity of the model depended on the success of the initialized landscape to accurately reflect contemporary forest composition and structure. The landscape initialization process [56] was informed by FIA data and produced a forest composition and structure that agreed with FIA data. Further, the evaluation of the Flammulated Owl model (Fig 2F) supports the validity of the initial LANDIS-II modeled landscape. The LANDFIRE and LANDIS-II informed models agreed (S3 and S4 Figs), therefore we surmised the LANDIS-II model timestep-0 outputs used to inform the American Three-toed Woodpecker model were reflective of the contemporary landscape. In addition, the FIA plots meeting the suitability criteria were generally associated with the areas identified by the model (S4 Fig). In this case, the validity of the suitability predictions would be a function of the model capturing the habitat use dynamics of the American Three-toed Woodpecker in the Northern Rockies Ecoregion and not the inputs used to inform it. The caveat to this assumption: the *snag density* predictions were not verifiable or comparable to the Flammulated Owl model, and they represent input data that is uncertain. We are not concerned as this variable is temporally dynamic and is not indicative of more long-term core habitat features further discussed.

Both avian models required inputs to be secondarily modeled from LANDIS-II simulated state variables. Canopy cover (Flammulated Owl), forest type (Flammulated Owl and American Three-toed Woodpecker), and snag density (American Three-toed Woodpecker) all required secondary modeling (derivative variable model) (Fig 2B). We implemented a RF model for each variable, because 1) we assumed complex and strong interactions among the predictor variables, which are notably handled by RF [87]; 2) prediction and not inference was our objective, making a machine learning process more advantageous [80]. The canopy cover model was the least robust, though this did not affect the Flammulated Owl habitat suitability map predictions (S1 File). RF has been used with success to predict canopy cover, but the predictor variables were more informative and based on inputs not derivable from LANDIS-II [88]. The secondary modeling introduces additional variability into the final potential and realized niche predictions, however models not informed by RF (Flammulated Owl) indicated the moderate error within the RF model (canopy cover) had a minimal effect on predictions (S1 File). Only the snag RF model could not be independently assessed, which is likely to have little effect on the information derived from the American Three-toed Woodpecker potential niche model because of the nature of the snag variable.

### LANDIS-II

The process of using LANDIS-II to produce spatially explicit inputs for use in other models has been done before [89], provides a means to simulate landscape level variables that are otherwise unavailable, and has its limitations. LANDIS-II estimates spatially explicit information that were unavailable over large spatial extents from alternative datasets. Integrating LANDIS-II allowed for the application of the American Three-toed Woodpecker model to the Northern Rockies Ecoregion. Data to inform the Woodpecker model were otherwise unavailable except for FIA plots, which would have limited the spatially explicit predictions possible via LANDIS-II. Using LANDIS-II in this integration provided opportunities, however it also presented limitations.

Restricted outputs from LANDIS-II presented a limitation to implementation of the proposed framework. This limited the scope of species and model types [90–92] that were feasible, because predictor variables (e.g., normalized burn ratio NBR) were not easily correlated to the LANDIS-II fire model outputs and modeling these was beyond the scope of this study. Management of species of conservation concern like the Lewis’s Woodpecker (*Melanerpes lewis*) [93] would benefit from model integration. However, the available models [92,94] were not easily transferable because of geographic and variable differences, specifically measures of landscape level fire effects beyond burned area (e.g., NBR) are not distinctly relatable to LANDIS-II fire severity outputs. However, with such modeled metrics provided (severity), it may be possible. Overall, the variable mismatches between avifauna-forest models and LANDIS-II limited the integration candidate set of ACN niche models.

A lack of suitable ACN niche models for integration can be addressed though development of in situ niche models based on habitat predictor variables easily sourced from forest landscape models [38,89,95]. Better integration through ACN predictor variable fit (Fig 2B) is possible through the use of alternative succession extensions like PnET [96] or forest landscape models like LANDIS-PRO [97], which can simulate leaf are index or density and basal area providing a better mechanism to estimate predictor variables like canopy cover [38]. Integrating LANDIS-II or other forest landscape models in the initial research development stages will likely minimize the constraints associated with variable mismatches and transferability [95].

### Habitat suitability maps

In Idaho, the Flammulated Owl is widely distributed in montane habitats but locally abundant with clustered spatial distribution of breeding sites [98]. This general pattern was exhibited in the predicted realized niche and the IDFG observation points (Fig 3). The increased probability of occupancy was generally not associated with observed locations and associated habitat use buffers (Table 3). The lower occupancy probabilities associated with the observed locations could result from uneven temporal recording intensity, spatial coverage, sampling effort, and temporal and spatial detectability, i.e., biased data [99]. It is likely these data are a function of these biases. Most of the data were incidental/opportunistic sightings spanning a 32-year period. Probability of detection is high for the Flammulated Owl among trained observers, though it is influenced by noise [51] and can decline significantly outside of the pair-bonding and incubation period [100]. The realized niche predictions may represent contemporary habitat suitability and the observation data may not represent contemporary habitat use because of less than perfect detectability and a likely shift in temporal habitat suitability over the 32-year period.

Alternatively, the niche suitability model may poorly approximate the realized niche in the novel study region because of resource use variations. Resource selection can vary between locations based on availability differences resulting in poor predictability of habitat use [101]. This can cause poor transferability of habitat suitability models [90]. The transferability assessment showed little difference between the model’s training region and the study area (S1 and S2 Figs) except in the covariance structure between ponderosa pine cover and two other variables (non-forest cover and canopy cover) (S2 Fig). However, prey availability, snag density, and stand density, which are associated with Flammulated Owl habitat, may influence resource selection, and are related to stand age and disturbance regime [102], which were not directly modeled. The age and forest structure underling the density of ponderosa pine and Douglas fir forest cover (included variables) is likely affecting habitat use due to resource availability differences between the model development and our study region. This is partially supported, because older stands were associated with the habitat use buffers. We did not assess if this represents a habitat use difference compared with the entire landscape, as this was beyond the scope of this research. The habitat suitability map is still useful in the management and protection of suitable habitat by focusing on those areas that are most suitable [51], or by focusing population trend and habitat suitability research needs [103] on areas of increased occupancy.

Assessing population abundance and trends for the American Three-toed Woodpecker is difficult; they are a highly irruptive species because of an association with newly (< 5 years old) burned forest patches [71]. Often associated with shifting food resources [104], irruptive species have irregular movement patterns making spatial predictions of habitat use also difficult [105]. We found little value in comparing the observed locations to the habitat suitability map, because of the irruptive characteristics and resulting sampling biases [99]. Any habitat suitability map will be highly temporally constrained and likely biased if disturbance characteristics resulting in shifting prey availability are not accurately modeled. However, land management and conservation activities will benefit from the spatial identification of habitats with key non-temporally sensitive niche characteristics (e.g., mature spruce forest). These landscape areas have the potential to confer suitable habitat after interacting with natural and anthropogenic disturbances. Focusing contemporary management and conservation activities on these core habitat areas is an investment in future potentially suitable habitat.

The niche attributes within the American Three-toe Woodpecker model that are temporally constrained but confer habitat suitability are snag density and time since last burn. The less temporally dynamic niche attributes are the mature/old growth forest, spruce forest cover, elevation, and areas devoid of harvest. The intersection of these niche attributes represents the core habitat areas to focus contemporary management activities. We found that these areas were aggregations of spatially fragmented forested blocks (Fig 5), which fragmentation and habitat loss are the main concerns for this species in Idaho [106]. Land management activities such as snag retention, fire management, and minimized timber harvest especially of mature/old growth forest would be best focused on these areas. In addition, future scenario modeling could evaluate the degree of impact of climate change and disturbance event interactions on the core habitat.

## Conclusion

The presented framework for the integration of ACN and forest landscape models based on the transfer of existing niche models is viable. Transferability was hindered by limitations such as model training region and study area landscape similarities in addition to forest landscape model output variables. We addressed these limitations through the criteria of selecting appropriate niche models, evaluating training and study area landscape similarities, and secondary modeling of niche model inputs from the forest landscape model outputs. The framework proved useful when niche models are not easily transferable to a landscape due to data constraints. LANDIS-II estimated spatially explicit landscape information (e.g., biomass distributions) that were unavailable from other datasets, and the framework included the process of comparing habitat suitability maps and underlying variables. This increased the application of avian niche models across a broad landscape improving habitat conservation information for land managers. Finally, this framework provides a process to ascertain species responses to climate change and management scenarios while providing forest ecosystem modelers with a means to account for wildlife species responses.

## Supporting information

S1 Fig The multivariate environmental similarity surface of the study area. Values <0 indicate locations that are novel and not present in the original region used to inform the niche suitability model. As a location approaches 100 the study area predictor values are all equal to the median value in the training region.
(TIF)

S2 Fig The covarience structure of the Flammulated Owl (Flam) realized niche model explanatory variables in the training region (BNF) and study area (NR). Intensity of the color or size of the pie indicate strength while red and blue indicate negative or positive relationships, respectively.
(TIFF)

S3 Fig Histogram of predicted occupancy probability values: A) Base model B) LANDIS-II model.
(TIFF)

S4 Fig The American Three-toed Woodpecker habitat suitability map with the corresponding FIA plots that meet the criteria of *very good* and *good* suitability. (TIF)

S1 File Walsh_methods_Paper_S1_file. (DOCX)
