# Supplementary figures and images for: An integration framework for linking avifauna niche and forest landscape models
Source: PLoS One. 2019 Jun 7;14(6):e0217299. doi: 10.1371/journal.pone.0217299 (PMC6555514; doi:10.1371/journal.pone.0217299)

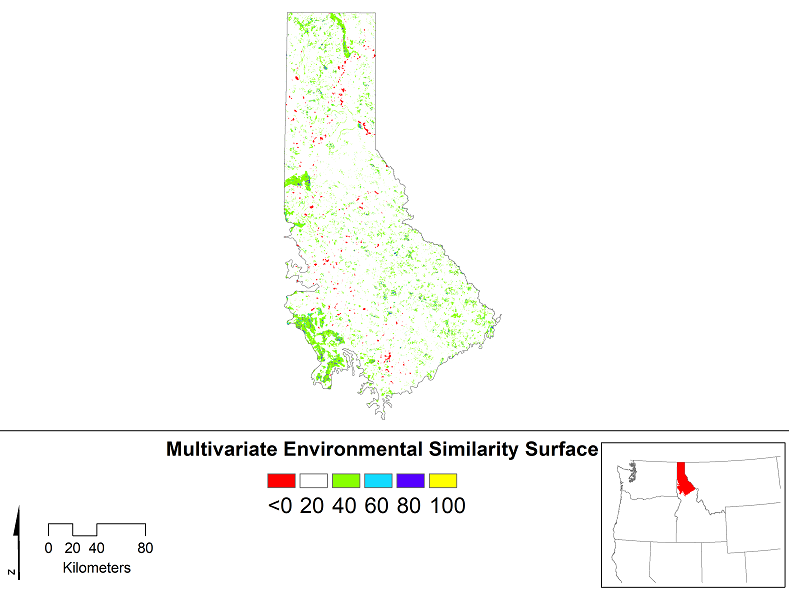

Supplement: S1 Fig — Values <0 indicate locations that are novel and not present in the original region used to inform the niche suitability model. As a location approaches 100 the study area predictor values are all equal to the median value in the training region. (TIF) [file pone.0217299.s001.tif]

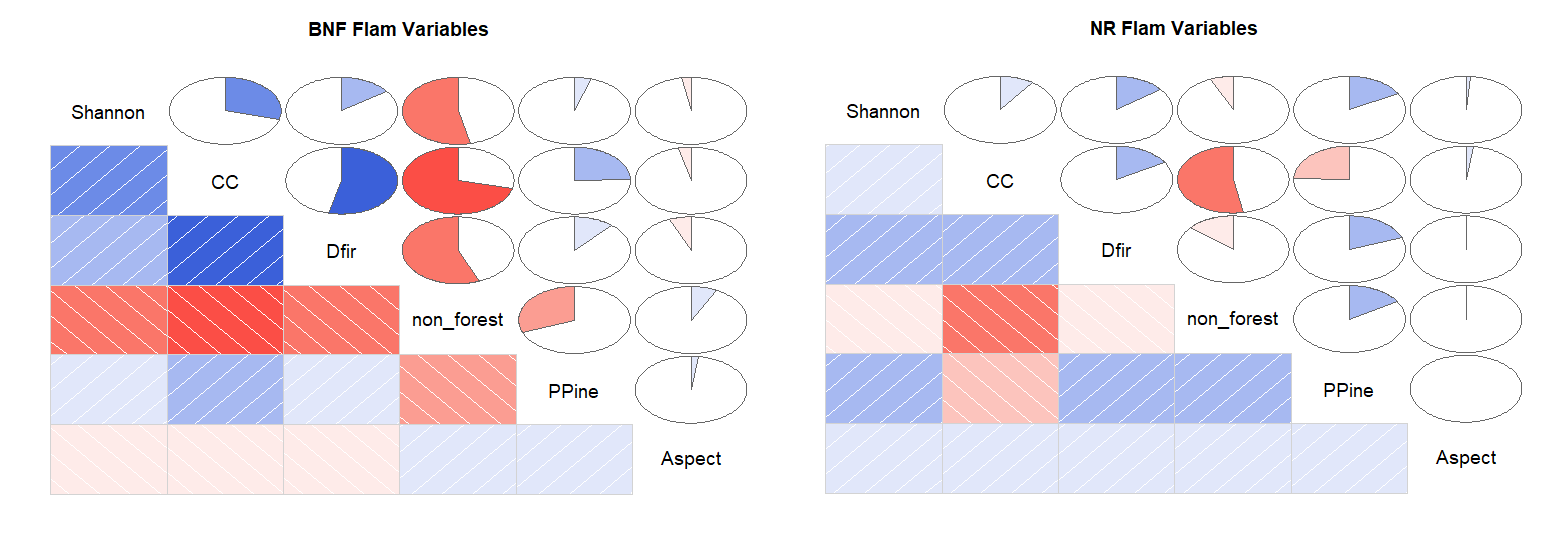

Supplement: S2 Fig — Intensity of the color or size of the pie indicate strength while red and blue indicate negative or positive relationships, respectively. (TIFF) [file pone.0217299.s002.tiff]

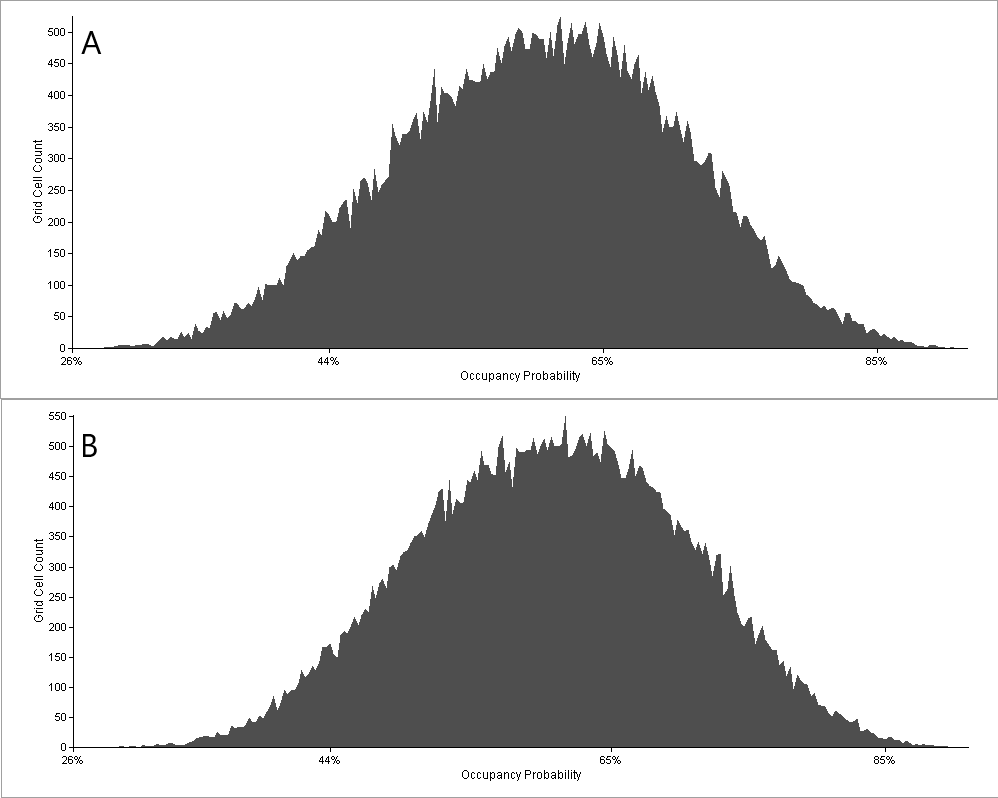

Supplement: S3 Fig — Histogram of predicted occupancy probability values: A) Base model B) LANDIS-II model. (TIFF) [file pone.0217299.s003.tiff]

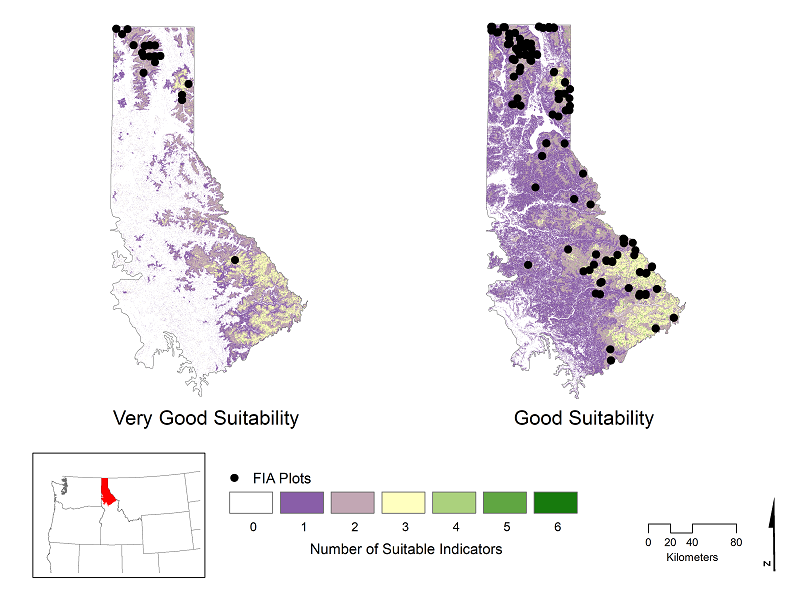

Supplement: S4 Fig — (TIF) [file pone.0217299.s004.tif]
