# Supplementary material for: An integration framework for linking avifauna niche and forest landscape models
Source: PLoS One. 2019 Jun 7;14(6):e0217299. doi: 10.1371/journal.pone.0217299 (PMC6555514; doi:10.1371/journal.pone.0217299)
Supplement: S1 File — (DOCX) [file pone.0217299.s005.docx]

**An integration framework for linking avian cavity nesting species niche models with forest landscape models.**

*Eric S. Walsh and Tara Hudiburg*

Supporting information file S1: Random Forest analyses, supplementary references, and figures.

# **S1.1 Random Forest analyses**

## *S1.1.1 General Random Forest implementation*

We conducted three Random Forest (RF) (1) classification analyses to predict the level of canopy cover (Flammulated Owl), the land cover type (Flammulated Owl and American Three-toed Woodpecker), and the snag presence (American Three-toed Woodpecker) of forested grid cells across the study area for the respective studies. The RF was implemented in Rstudio (2) with R (v. 3.5.1) (3) using the *randomForest* package (4). The algorithm is optimized via three parameters *ntree*, *mtry*, and *nodesize* (Table S1.3.1). The parameter *ntree* is the number of trees grown from a bootstrapped sample and was determined based on a stabilized prediction error; *mtry* is the number of predictors randomly tested at each node, which was determined using the randomForest’s *tuneRF* function; *nodesize* is the minimal size (i.e., cases) of the terminal node. The *nodesize* was set to values >1 to improve model computation given the large sample size, which has minimal effect on accuracy for large datasets (1). Random Forest produces an unbiased error estimate (out-of-box (OOB) error), enabling model validation without an independent dataset (1,5).

## *S1.1.2 Canopy cover analysis*

We used a RF classification analysis to predict the level of canopy cover of each forested grid cell across the study area. There were four canopy cover classes corresponding to the parameterization described in (6). We trained the model using 200 m gridded data of the Contiguous U.S. Biomass Map (biomass) (7), elevation (8), aspect, slope, and landform class (a derivative of topographic position index) as predictor variables with the LANDFIRE 2008 Forest Canopy Cover (9) data as the dependent variable. Aspect, slope, elevation, and landform index were included because of their associations with ecosystem characteristics (10,11). Landform index is a classification system based on the topographic position index (12,13) (derived from elevation and slope), which indicates the type of terrain (e.g., canyon, mid slope, or ridge) present in each grid cell. The response variable canopy cover class was derived from the LANDFIRE data aggregated into four canopy cover classes (1 = 0-10%, 2 = 11-40%, 3 = 41-70%, and 4 = 71-100%) with the category’s midpoint value assigned to a grid cell (6). The canopy cover RF model OOB error was 45.1% with the most accurately predicted class being *medium* and least being *high* (Table S1.3.3). The strongest predictor of canopy cover class was biomass (Fig S1.4.1).

## *S1.1.3 Land cover type analysis*

We used a RF classification analysis to predict the 11 cover classes corresponding to the parameterization described in (6). We trained the model using 200 m gridded data of elevation (8), aspect, slope, landform class, soil parameters (field capacity, available water, and wilting point) (14), and tree species biomass totals as predictor variables with the LANDFIRE Existing Vegetation Type data (15) as the dependent variable. Elevation, aspect, slope, and landform have been used in similar modeling approaches (16,17). Soil characteristic were included because of their association with forest cover types of northern Idaho from initial modeling (data not shown) and association with biomass (18). The tree species biomass totals were estimated from the LANDIS-II spin-up models. These data were included because they are outputs of LANDIS-II allowing for model inclusion in future scenario runs. However, unlike the canopy cover model that trained on a dataset independent of LANDIS-II outputs, there was no alternative dataset that provided the same level of species-biomass totals per grid cell. However, these data were directly informed from Forest Inventory Analysis data (19) using a landscape “seeding” process (20). The final land cover map used in the Flammulated Owl model was based on only three land cover types (Douglas fir, ponderosa pine, and vegetative non-forest) (6), therefore we were most concerned with the prediction of these cover types. The land cover RF model OOB error was 9.4% with the (Table S1.3.4). The strongest predictors of land cover type were Douglas fir (Dfir), grand fir (Gfir), and elevation (DEM) (Fig S1.4.2).

## *S1.1.4 Snag presence analyses*

We used a RF classification analysis to predict the snag presence. Each data point in the RF training set represented a Forest Inventory Analysis (FIA) plot within the study area coded as the appropriate snag density being present/absent. The covariate data associated with each data point included elevation (8), aspect, slope, average forest age, and total aboveground live biomass (AGB). The forest age and AGB were derived from the FIA plot data. The snag RF model OOB error was 25.7% with the (Table S1.3.5). The strongest predictors of land cover type were AGB (AboveLiveB) and forest age (AGE) (Fig S1.4.3). A RF model with aboveground dead biomass (an alternative to live biomass) may have provided a better fit, however the LANDIS-II succession extension we implemented did not output spatially explicit maps of aboveground dead biomass.

# **S1.2 Supporting References**

1. Breiman L. Random forests. Mach Learn [Internet]. 2001;45(1):5–32. Available from: http://link.springer.com/article/10.1023/A:1010933404324

2. RStudio Team. RStudio: Integrated Development Environment for R [Internet]. Boston, MA; 2018. Available from: http://www.rstudio.com/

3. Team R Core. R: A Language and Environment for Statistical Computing [Internet]. Vienna, Austria; 2018. Available from: http://www.r-project.org/

4. Liaw A, Wiener M. Classification and regression by randomForest. R news. 2002;2(3):18–22.

5. Siroky DS. Navigating Random Forests and related advances in algorithmic modeling. Stat Surv. 2009;3:147–63.

6. Scholer MN, Leu ML, Belthoff JR. Factors Associated with Flammulated Owl and Northern Saw-Whet Owl Occupancy in Southern Idaho. J Raptor Res [Internet]. 2014;48(2):128–41. Available from: http://www.bioone.org/doi/abs/10.3356/JRR-13-00049.1

7. Blackard JA, Finco M V., Helmer EH, Holden GR, Hoppus ML, Jacobs DM, et al. Mapping U.S. forest biomass using nationwide forest inventory data and moderate resolution information. Remote Sens Environ. 2008;112(4):1658–77.

8. U.S. Geological Survey (USGS) EDC. National Elevation Dataset for Idaho (1 arc second | 30-meter) [Internet]. Sioux Falls, SD: U.S. Geological Survey; 1999 [cited 2015 Jun 2]. Available from: http://cloud.insideidaho.org/webMaps/flash/tiledownload/index.html?collection=elevation&layerName=1999_30m_Idaho

9. LANDFIRE. LANDFIRE: Forest Canopy Cover. LANDFIRE 1.2.0. 2008.

10. Bale CL, Williams JB, Charley JL. The impact of aspect on forest structure and floristics in some Eastern Australian sites. For Ecol Manage. 1998;110:363–77.

11. Suchar VA, Crookston NL. Understory cover and biomass indices predictions for forest ecosystems of the Northwestern United States. Ecol Indic. 2010;10(3):602–9.

12. Weiss A. Topographic position and landforms analysis. In: ESRI User Conference. San Diago,CA; 2001.

13. Jenness J, Brost B, Beir P. Land facet corridor designer [Internet]. 2013. p. 10. Available from: www.corridordesign.org

14. Soil Survey Staff Natural. Web Soil Survey [Internet]. Natural Resources Conservation Service. 2015 [cited 2015 Feb 6]. Available from: http://websoilsurvey.nrcs.usda.gov/

15. LANDFIRE. LANDFIRE: Existing Vegetation Type. LANDFIRE 1.4.0. U.S. Department of Agriculture and U.S. Department of the Interior; 2014.

16. Moisen GG, Frescino TS. Comparing Five Modelling Techniques for Prediction Forest Characteristics. Ecol Modell. 2002;157:209–25.

17. Hörsch B. Modelling the spatial distribution of montane and subalpine forests in the central Alps using digital elevation models. Ecol Modell. 2003;168(3):267–82.

18. Piedallu C, Gégout JC, Bruand A, Seynave I. Mapping soil water holding capacity over large areas to predict potential production of forest stands. Geoderma [Internet]. 2011;160(3–4):355–66. Available from: http://dx.doi.org/10.1016/j.geoderma.2010.10.004

19. U.S. Department of Agriculture FS. Forest inventory and analysis national program - data and tools - FIA data mart, FIADB Version 4.0. Washington, DC; 2015.

20. Dijak W. Landscape Builder: software for the creation of initial landscapes for LANDIS from FIA data. Comput Ecol Softw. 2013;3(2):17–25.

# **S1.3 Supporting Tables**

Table S1.3.1. Random Forest parameters used to optimize performance

| **Parameter** | **Canopy Cover** | **Forest Cover** | **Snag Presence** |
| --- | --- | --- | --- |
| ntree | 1000 | 1000 | 10000 |
| mtry | 2 |  | 1 |
| nodesize | 5 |  | 1 |

Table S1.3.2. Explanatory variables for the canopy cover Random Forest model.

| **Variable** | **Rational** | **Value** |
| --- | --- | --- |
| aspect | niche differentiation | degrees |
| elevation | niche differentiation | elevation in meters |
| slope | niche differentiation | degrees |
| biomass | proxy for tree density and Leaf Area Index | grams/m^2^ |
| landform | niche differentiation | index value |

**Table S1.3.3.** The confusion matrix of the Random Forest canopy cover classification model with omission error.

| Prediction | Validation | | | | | |
| --- | --- | --- | --- | --- | --- | --- |
|  |  | High | Low | Medium | Medium-Low | **Omission Error** |
|  | High | 9886 | 289 | 18739 | 1577 | 0.68 |
|  | Medium | 6860 | 2149 | 62336 | 11714 | 0.25 |
|  | Medium-Low | 892 | 5311 | 21885 | 18208 | 0.61 |
|  | Low | 199 | 8361 | 4199 | 7395 | 0.59 |
|  | **Commission Error** | 0.46 | 0.87 | 0.80 | 0.81 |  |

| Validation | | | | | | | | | | | | | |
| --- | --- | --- | --- | --- | --- | --- | --- | --- | --- | --- | --- | --- | --- |
| Prediction |  | Anthropogenic | Non-Forest | Riparian | Aspen | Douglas-fir | Lodgepole | Ponderosa Pine | Larch | Mix-conifer | Spruce-Fir | Barren | **Omission Error** |
|  | Anthropogenic | 8953 | 1833 | 524 | 0 | 0 | 57 | 149 | 0 | 0 | 1 | 0 | 0.22 |
|  | Non-forest | 1711 | 18081 | 1818 | 2 | 0 | 430 | 238 | 1 | 0 | 1098 | 0 | 0.23 |
|  | Riparian | 463 | 1924 | 4343 | 2 | 0 | 37 | 61 | 2 | 0 | 0 | 0 | 0.36 |
|  | Aspen | 2 | 29 | 2 | 7 | 0 | 0 | 0 | 0 | 0 | 0 | 0 | 0.83 |
|  | Douglas-fir | 15 | 38 | 4 | 0 | 44739 | 10 | 123 | 0 | 1108 | 246 | 0 | 0.03 |
|  | Lodgepole | 42 | 767 | 106 | 0 | 0 | 1492 | 11 | 0 | 0 | 483 | 0 | 0.49 |
|  | Ponderosa Pine | 226 | 474 | 237 | 0 | 450 | 6 | 1918 | 0 | 38 | 3 | 0 | 0.43 |
|  | Larch | 6 | 58 | 9 | 0 | 0 | 0 | 0 | 2 | 0 | 4 | 0 | 0.97 |
|  | Mix-conifer | 5 | 55 | 4 | 0 | 865 | 1 | 10 | 0 | 56447 | 316 | 0 | 0.02 |
|  | Spruce-Fir | 4 | 255 | 0 | 0 | 125 | 10 | 0 | 0 | 306 | 27120 | 0 | 0.03 |
|  | Barren | 1 | 75 | 11 | 0 | 0 | 1 | 0 | 0 | 0 | 5 | 1 | 0.99 |
|  | **Commission Error** | 0.22 | 0.23 | 0.38 | 0.36 | 0.03 | 0.27 | 0.23 | 0.6 | 0.03 | 0.07 | 0 |  |

**Table S1.3.4.** The confusion matrix of the Random Forest land cover classification model.

**Table S1.3.5.** The confusion matrix of the Random Forest snag classification model.

| Validation | | | | |  |
| --- | --- | --- | --- | --- | --- |
| Prediction |  | No Snags | Snags | **Omission Error** | |
|  | No Snags | 681 | 152 | 0.18 | |
|  | Snags | 216 | 374 | 0.36 | |
|  | **Commission Error** | 0.24 | 0.29 |  | |

# **S1.4 Supporting Figures**

**
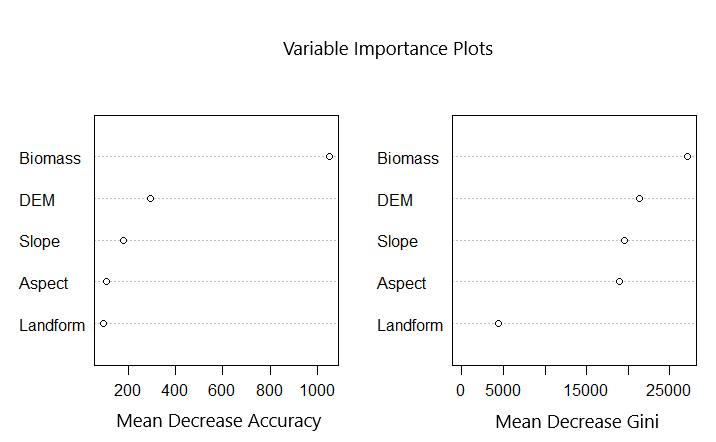
**

Figure S1.4.1. The variable importance plots for the canopy cover Random Forest analysis.


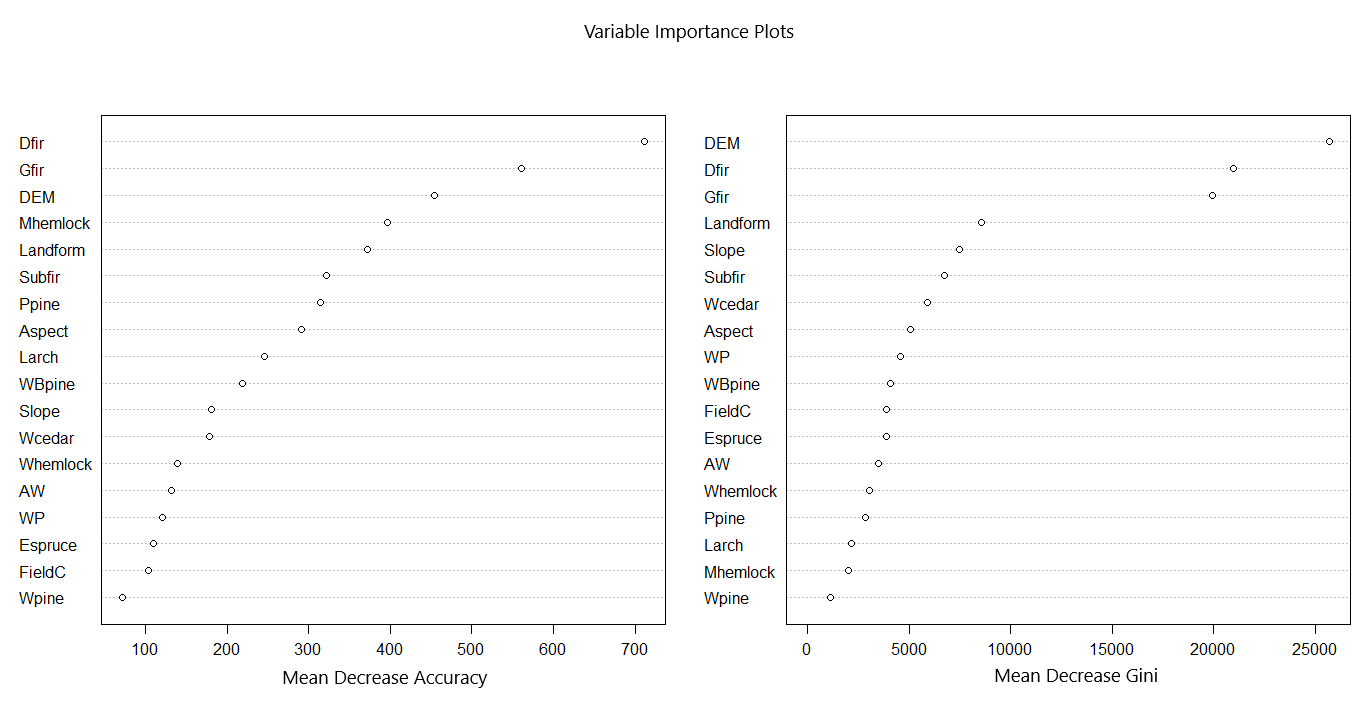


## **Figure S1.4.2.** The variable importance plots for the land cover Random Forest analysis


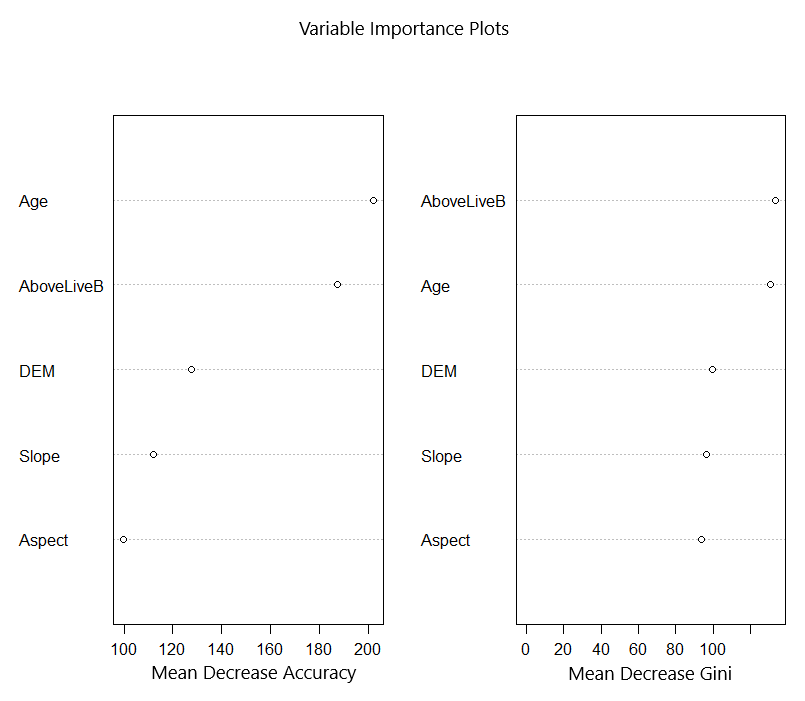


## **Figure S1.4.3.** The variable importance plots for the snag Random Forest analysis
